# Implementation of the frailty assessment to improve liver transplant outcomes

**DOI:** 10.1007/s40520-022-02111-1

**Published:** 2022-04-05

**Authors:** Mattia Corradi, Chiara Mazzarelli, Matteo Cesari, Raffaella Viganò, Luca Saverio Belli

**Affiliations:** 1 Scuola di Specializzazione in Malattie dell’Apparato Digerente, University of Milan, Milan, Italy; 2 Hepatology and Gastroenterology Unit, ASST Grande Ospedale Metropolitano Niguarda, Milan, Italy; 3 Department of Clinical Sciences and Community Health, University of Milan, Milan, Italy

**Keywords:** Frailty, Liver Transplantation, Long term survivors, Improving outcomes, Frailty Index

## Abstract

The majority of patients undergoing Orthotopic Liver Transplantation (OLT) have increased in age, therefore chronological age may have become an unreliable parameter for supporting clinical decisions. The age-related deficit accumulation model measuring frailty proposed by Rockwood et al., may propose an alternative in providing an estimate of an individual’s biological age. No Frailty Index (FI) tailored specifically for OLT patients exists to date. Forty-three consecutive OLT patients with ≥ 20 years of survival with a functioning graft were included in our study. The FI was computed taking to account 39 items (FI-39), meeting the standard criteria for internal validation. Endpoints were polypharmacy, and recent Emergency Room admission. The mean age of our population was 69 (sd 9) years. The mean FI-39 was 0.23 (sd 0.1). The FI-39 was associated with polypharmacy [odds ratio (OR) 1.13; Confidence interval (95%CI) 1.03–1.24; *p* = 0.01], and recent Emergency Room admission [beta coefficient + 1.98; 95%CI + 0.26, + 3.70; *p* = 0.03], independent for age and sex. This study demonstrates that an FI can be derived from data collected during routine clinical follow-up and allows for improved differentiation related to the OLT clinical complexity in OLT patients, independent of chronological age. This may lead to the adoption of FI-39 to improve personalized OLT patient care.

## Introduction

 The scientific community is identifying surrogate markers for chronological age in geriatric patients to improve care and interventions. This has led to the concept of frailty. It is a condition characterized by a reduction in the individual’s physiological reserves and increased risk of adverse health outcomes [1]. This is also seen in the Orthotopic Liver Transplantation (OLT) patient although to date, clinical practice uses chronological age in this patient group.

OLT represents the gold standard in the treatment of acute liver failure, end-stage liver diseases, and hepatocellular carcinoma. It is a complex intervention with burden of early and late complications. In addition, the mean age of the OLT population has increased in recent years. This is due to the increase in recipient age at time of transplant, the increase in survival rate provided by the improvement in immunosuppression protocols, and a decreased mortality related to comorbidities [2]. However, despite this, surgical outcomes, chronic immunosuppression, and related comorbidities contribute to the accelerated aging process in OLT long-term survivors. This aging process has commonly been quantified in chronological age despite its limitations.

This present study aims to generate and validate a novel Frailty Index, designed in accordance with the theoretical model proposed by Mitnitski [3], standardized by Searle [4], the FI-39 index for OLT patients. The robustness will be internally verified; the predictive capacity of the FI-39 index will also be validated for specific clinical outcomes, such as recent Emergency Room (ER) admissions and polypharmacy.

## Materials and methods

**Fig. 1:**
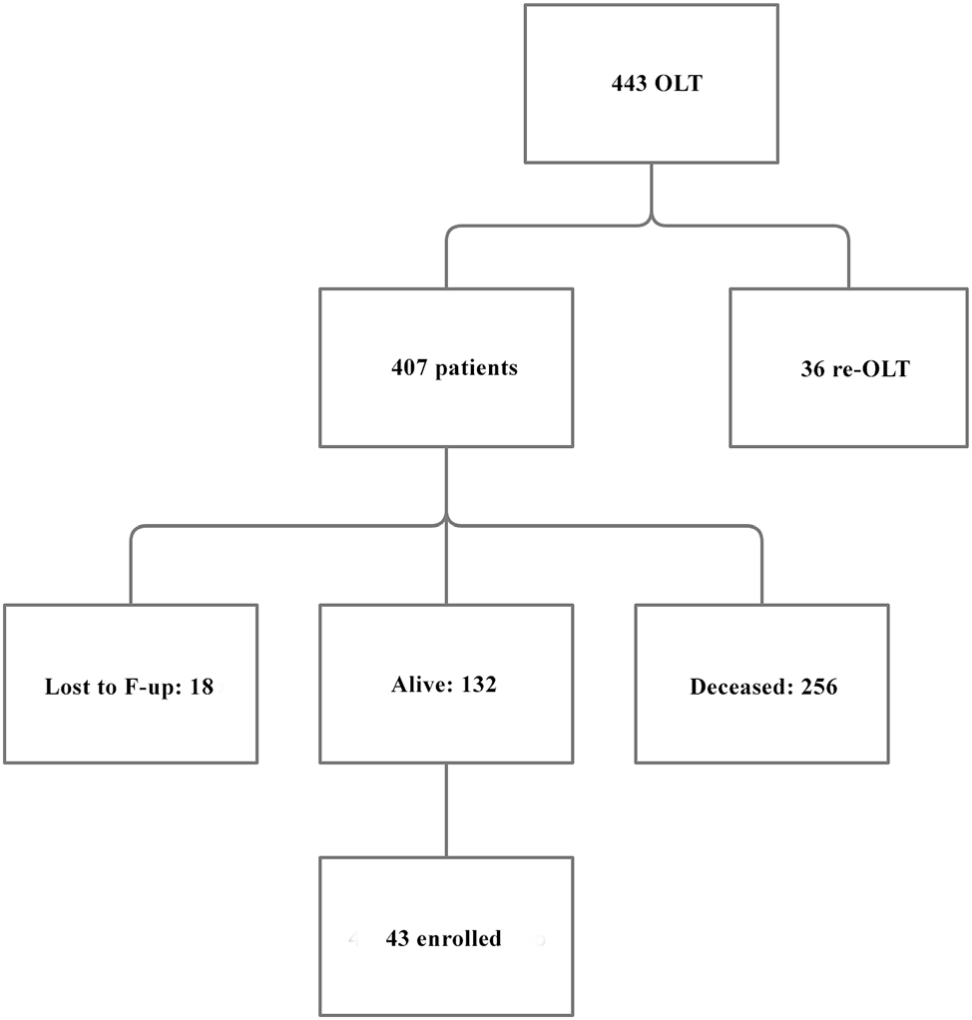
Enrollment of the patients included in our study

An observational, cross-sectional study was conducted on forty-three consecutive OLT patients with ≥ 20 years of survival with a functioning graft, referring to the Liver Transplant Centre of the ASST Grande Ospedale Metropolitano Niguarda (Milan, Italy) (see Fig. 1).

A 39-item FI was generated using clinical information, including signs, symptoms, diseases, disabilities, biological abnormalities, and directly retrieved from medical records (see Table 1). The data were collected during routine examination in the post-OLT ward. Most of the variables were already taken into consideration in the usual routine (i.e., blood values, vital signs); some others (i.e., handgrip) have been measured expressly for building up our index. The collection of these data was performed by a study technician taking between 5 and 10 min for each patient.

**Table 1:** List of the 39 health deficits composing the FI-39

Health deficits
AST abnormality (> 40 U/L)
ALT abnormality (> 45 U/L)
Bilirubin abnormality (> 1.0 mg/dL)
Hemoglobin abnormality (< 14.1 g/dL)
Creatinine abnormality (> 1.17 mg/dL)
Gamma-glutamyl transferase abnormality (> 50 mg/dL)
Alkaline phosphatase abnormality (> 129 mg/dL)
Systolic blood pressure abnormality (< 100 or > 140 mmHg)
Diastolic blood pressure abnormality (< 60 or > 90 mmHg)
Muscle weakness assessed using dynamometer
Obesity
Need help for eating
Need help for housekeeping
Need help with finances
Disability in transportation
Balance disorders
Mobility disability
Memory complaints
Self-reported insomnia
Hearing impairment
Vision impairment
Tremors
Osteoporosis
Urinary incontinence
Thyroid disease
Gastrointestinal disease
Renal disease
Skin disease
Biliary tract disease
Chronic respiratory disease
Cerebrovascular disease
History of myocardial infarction
Signs of cardiac decompensation
Cardiac arrhythmias
Diabetes
Cancer
Osteoarthritis
Asthenia
History/family history of neurodegenerative diseases

The FI-39 was then calculated as the ratio between the number of the health deficits present in the individual patient (presence of the deficit = 1, absence of the deficit = 0) with the number of considered deficits, being 39 in the study. Thus, an FI could theoretically range between 0 and 1, complete absence of deficits and all deficits are present, respectively [4].

To verify whether the generated FI-39 was able to capture the clinical vulnerability of the individual, two outcomes were defined:

1. A recent ER admission that is occurred over the previous 12 months.
2. The presence of polypharmacy, defined as the simultaneous use of four or more medications per day [5].

The study sample was described using prevalences and means (with standard deviations) for the variables of interest. Pearson’s correlation analysis was performed to test the relationship between FI-39 and chronological age. Unadjusted and adjusted logistic regression models were run to measure the association of the study outcomes with the independent variable of interest. A *p* value lower than 0.05 (two-tailed) was considered to define statistical significance. Statistical analysis was conducted using IBM SPSS version 26 for Windows.

## Results

The study sample (*n* = 43) included 29 men and 14 women, mean age at enrollment 69 years (standard deviation, SD 9). Patient comorbidities included, renal disease (47%); hypertension (47%); and osteopenia/osteoporosis, grouped together, (42%); were the most prevalent. This was predictable, given the long-term immunosuppressive treatment [2].

The diseases that lead to transplant in our population were mostly virus-related cirrhosis (34 patients with HCV being the most present; among these, 13 patients developed a hepatocellular carcinoma). The remaining 9 patients affected by cirrhosis were of cryptogenetic origin or biliary.

Mean daily drug burden was 7 (SD 4), including immuno-suppressants with monotherapy most common (22 patients, 53%), whereas the remaining patients used a 2 drug regimen; only one individual was on a triple therapy. Polypharmacy (number of drugs ≥ 4) was present in 24 patients (57.1%). ER access was seen as at least 1 visit in the previous 12 months in 13 patients (30.2%) (Table 2).

**Table 2:** Patient Demographics and clinical characteristics

	*N* (%)
Gender male	29 (67%)
Mean age	69 (sd 9)
BMI < 18,5 o > 25 (kg/m^2^)	21 (49%)
Kidney disease	20 (47%)
Osteopenia/osteoporosis	18 (42%)
Diabetes	11 (26%)
Hypertension	20 (47%)
No. medications taken	7.0 (sd 4)
1 immunosuppressant drug taken	23 (53%)
2 immunosuppressant drugs taken	20 (44%)
3 immunosuppressant drugs taken	1 (3%)
At least one access to the ER during the past 12 months	13 (30%)

**Fig. 2:**
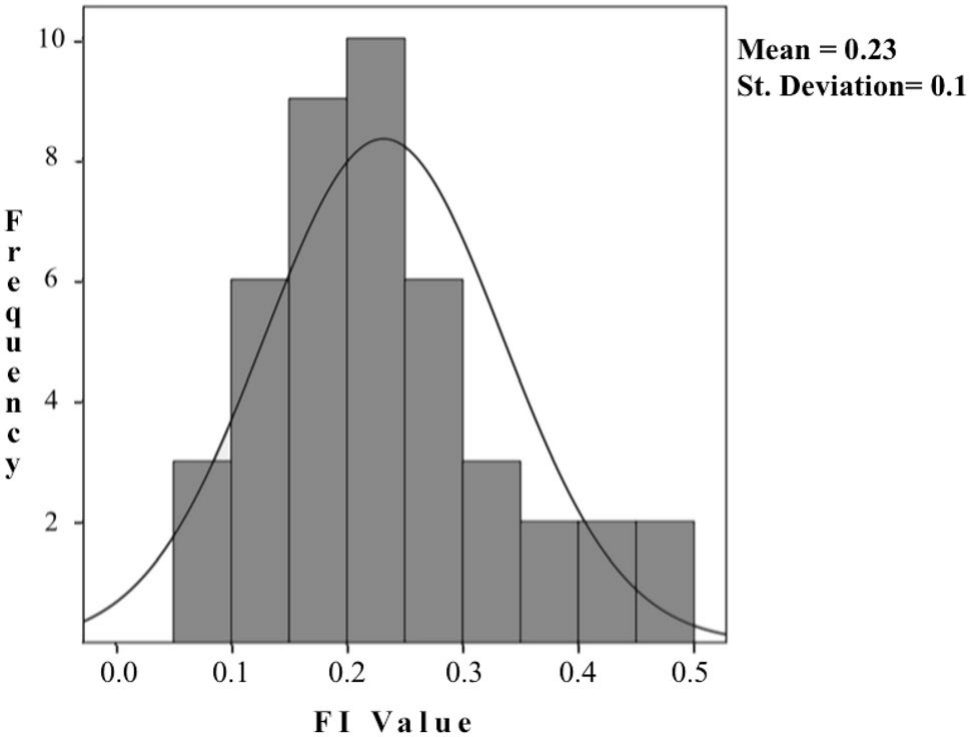
Distribution of the FI-39. No participant had a FI value equal to or higher than 0.7

The FI-39 mean value was 0.23 (SD 0.10; range 0.06–0.47; median 0.21) having a relatively normal distribution (see Fig. 2).

**Fig. 3:**
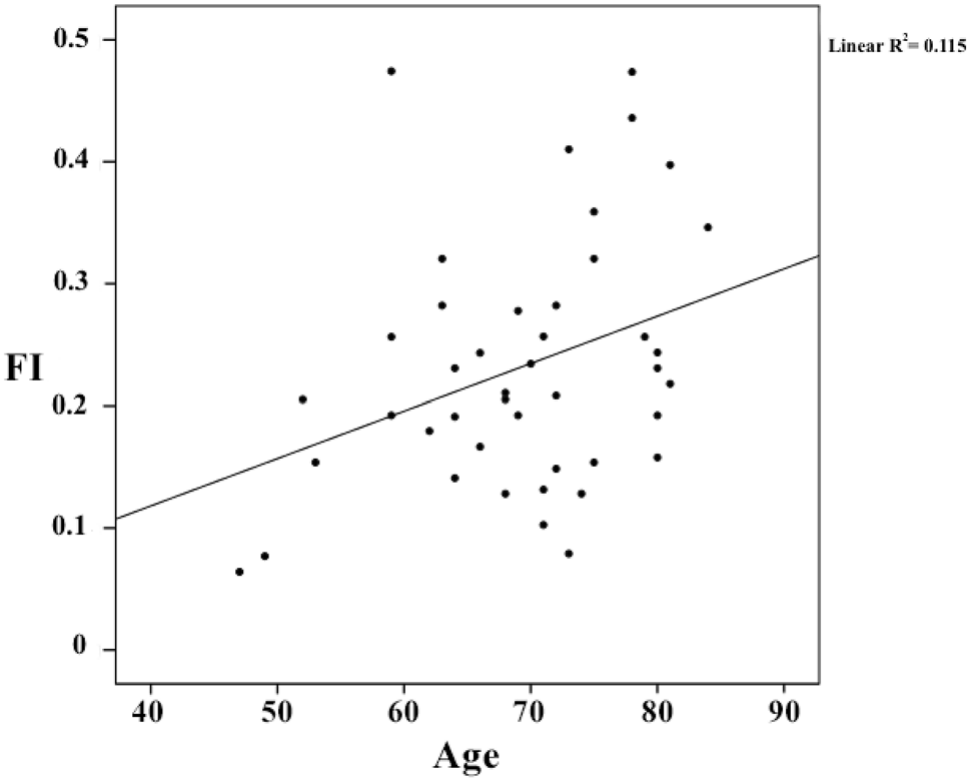
Scatterplot showing the linear relationship between the FI-39 and chronological age

Pearson’s analysis showed a statistically significant and direct correlation between FI and age (*r* = 0.34, *p* = 0.03) (see Fig. 3).

A significant positive relationship was reported between FI-39 and drug burden using linear regression models, even after adjustment for age and sex. Even excluding immuno-suppressants, our results were not affected (Beta coefficient + 17.34; *p* = 0.001). Consistent results were reported using adjusted and unadjusted logistic regression models, adopting polypharmacy as the dependent variable (see Table 3).

**Table 3:** Correlation between FI-39 and drug burden and between FI-39 and polypharmacy

	β	*CI* 95%	*p*
FI-39–drug burden	+ 18,332	8,057 ; 28,607	0,001
FI-39–drug burden (adjusted for age and sex)	+ 17,983	6,662 ; 29,334	0,003

	OR	*CI* 95%	*p*
FI-39–polypharmacy	1,13	1,030 ; 1,240	0,01
FI-39–polypharmacy (adjusted for age and sex)	1,123	1,014 ; 1,245	0,026

The unadjusted linear regression model exploring the relationship between the FI-39 and the number of visits to the ER, reported a significant association. However, there was a lack of statistical significance when the model was adjusted for age and sex as covariates, although without notable change in the beta coefficient (see Table 4).

**Table 4:** Correlation between FI-39 and ER admissions

	β	CI 95%	*p*
FI-39–ER admission in the last years	+ 1,979	0,263; 3,696	0,025
FI-39–ER admission in the last years (adjusted for age and sex)	+ 1,663	− 0,194; 3,521	0,78

Considering the cause that leads to transplant, no differences statistically significative were found in our FI. No correlation either was found between FI and yrs of post OLT (mean 23 years, SD 3) using Spearman’s correlation (*r* = 0.014; *p* = 0.93).

## Discussion

In literature, many assessment tools have been proposed to measure frailty [6–9], some of them also in the field of liver transplantation. Nevertheless, there is a relative lack of instruments that comprehensively and holistically measure the complexity of frailty in this subgroup of patients. An example is the tool developed by Lai et al. [10] focusing on the physical domain of the individual, by capturing the frailty phenotype proposed by Fried et al. [11], with Lai et al. further recognizing the limitations for use in OLT patient follow-up [12]. Based on this, we devised the novel FI-39 for OLT patients.

The FI-39 was generated and validated, both internally and in association with clinically relevant outcomes. The internal validation of the FI-39 was done using its association with chronological age, and the absence of patients with an FI-39 score > 0.7, which is a threshold that has been frequently indicated as incompatible with life [13].

Our findings confirm the possibility of directly creating this surrogate of biological age from medical records and its predictive capacity for endpoints of clinical interest, polypharmacy and recent ER admissions.

An FI is seen to be more reliable than just using the criteria of chronical age as it gives an objective numeric parameter mirroring the biological age of the patient. Furthermore, limiting the weight that single diseases or conditions may play in the decision-making process. Whereby an FI is considered quantitative, not qualitative, as the number of variables, not the type of variables included, defines it robustness. When the clinical information is sufficiently multidimensional, not too focused on organ or disease, it is sufficient to have a minimum of 20 health deficits to generate a reliable FI (although 30 are recommended to achieve a certain level of robustness) [4]. Thus, as data collection is routine in clinical setting albeit time-consuming, an FI can be easily generated by relying on standard assessment criteria collected during routine clinical follow-up [4].

Despite there being numerous publications with short- or medium-term follow-up, very few studies have analyzed the long-term survivors who received a liver transplant ≥ 20 years ago to date. This study aims to focus on this subset of patients for two main reasons:

–To have a population exposed to immuno-suppressants’ side effect for a time long enough to have an impact on the aging process.
–As the FI in designed in the geriatric field, we wanted to analyze a geriatric population given the mean age at OLT of about 40–50 years.

Extending our study to every OLT recipient followed up in the center would have implied a much more time-consuming data collection and analysis we could not afford at the moment with the COVID-19 pandemic having forced the closure of our recruitment. This resulted in a possible limitation of our study—having a small sample with strict inclusion criteria—even if our results were statistically significative.

Furthermore, the cross-sectional nature of the analysis does not allow for establishing any cause-effect relationship. As we performed a retrospective analysis, it would have been impossible to date back to the same health deficits. Obviously, it would be interesting to perform a follow-up on these patients using the same tool to evaluate their health status conducting a prospective analysis.

In this context, the demonstrated association with clinical outcomes has to be carefully considered as the attempt to validate FI for a clinically overt status of frailty.

In conclusion, an FI is a valid and well-established instrument for estimating an individual’s biological age. A shift in the clinician's forma mentis is required by the demographic phenomenon leading to a steady increase in the mean age in the various clinical services, independent of the specialty [14]. Our FI-39 in its application is feasible and results reproducible in the field of OLT, as demonstrated in the present study. The adoption of FI-39 in the clinical OLT setting is a novel tool that may aid transplant clinicians in improving long-term outcomes in OLT patients, providing a complex understanding of the patient, with increased personalized patient care. Further studies are needed in applying the FI-39 to all OLT patients irrespective of years of post OLT.
